# Results of End-To-Side Hypoglossal-Facial Nerve Anastomosis in Facial Paralysis after Skull Base Surgery

**DOI:** 10.22038/ijorl.2019.36294.2194

**Published:** 2020-05

**Authors:** Sasan Dabiri, Mohammadtaghi Khorsandi Ashtiani, Melorina Moharreri, Zahra Mahvi Khomami, Ali Kouhi, Nasrin Yazdani, Pedram Borghei, Kayvan Aghazadeh

**Affiliations:** 1 *Otorhinolaryngology Research Center, Amir Alam Hospital, Tehran University of Medical Sciences, Tehran, Iran.*

**Keywords:** Facial nerve paralysis, Hypoglossal-facial nerve anastomosis, Rehabilitation

## Abstract

**Introduction:**

The primary aim of facial reanimation surgery is to restore tone, symmetry, and movement to the paralyzed face. Hypoglossal-facial end-to-side anastomosis provides satisfactory facial reanimation in the irreversible proximal injury of the facial nerve. This study discussed the facial function results of end-to-side anastomosing of hypoglossal nerve to facial nerve when the injury occurred during skull base surgery.

**Materials and Methods:**

The present study enrolled a total of 10 patients who underwent end-to-side hypoglossal-facial nerve anastomosis after facial nerve paralysis due to skull base surgery. The data of the patients were gathered from hospital records, pictures, and movies during the 18 months of follow-up.

**Results:**

At the 18 months of follow-up, seven (70%) and three (30%) patients were reported with grades III and IV of the House-Brackmann scoring system, respectively. In total, out of the seven grade III patients, six subjects underwent early anastomosis (within the first year of the paralysis). On the other hand, among patients with grade IV, two subjects had late anastomosis.

**Conclusion:**

It seems that early end-to-side hypoglossal-facial anastomosis can be a favorable surgical option with good facial function results for reanimating the facial function of patients with facial paralysis following skull base surgery.

## Introduction

When the disruption of the nerve occurs, the elasticity of the endoneurium causes the retraction of the injured ends. Nerve vasculature is traumatized, and inflammation with secondary activation of fibroblasts leads to the formation of a dense scar at the site of injury. The diameter of the proximal side is generally reduced due to the functional loss of connection between the end-organ muscle and nerve Schwann cells. Nerve potentials at the distal side of the injury are present until total degeneration happens. Therefore, the evaluation of nerve function and severity of the injury should not be performed by electrophysiological studies during the first several weeks.

Wallerian degeneration begins after injury in hours, and both neuron and myelin sheath are affected. The aforementioned process is usually completed by 2 months. The endoneurial tubes shrink and their sheath thickens due to collagen deposition. The regeneration of the cut axon toward its correct muscle fibers depends on guidance by the basal Schwann cell lamina (i.e., axonotmesis) or grafted basal lamina (i.e., neurotmesis). Nerve regeneration continues for months to years (1).

The primary aims of facial reanimation surgery are the restoration of the tone, symmetry, and movement of the paralyzed face and subsequent improvement in the quality of life. There are many reliable techniques available to achieve these aims, and treatment plans can be tailored to the patient's situation (2,3). 

Classic hypoglossal-facial neurorrhaphy has been considered an effective strategy for the reanimation of the paralyzed face. It is performed when a proximal facial nerve segment is unavailable for reconstruction, such as after ablative tumor surgery or trauma involving the cranial base (4). 

End-to-side hypoglossal-facial nerve anastomosis (HFA) results in satisfactory facial reanimation after irreversible proximal nerve injury. In this technique, the epineurium of the distal end of the facial nerve and proximal end of the hypoglossal nerve are coapted together through a window created in the hypoglossal nerve (5-7). By the end-to-side technique, the major complications of the complete transaction of the 12^th^ cranial nerve, such as the atrophy of the tongue and problem with swallowing in the oral phase, could be preventable (5,7-11). 

The time between facial nerve injury and HFA is a significant factor for the ultimate facial nerve outcome (10,12). The HFA during the first 2 years following an injury to the facial nerve could be accompanied by the best outcomes (9,13). The identification of a surgical technique with the best results of facial reanimation for proximal facial nerve injury is still a debate. Han and the colleagues demonstrated that facial function outcomes in split type HFA are more favorable than those reported for end-to-side hypoglossal-facial anastomosis (14). However, some authors believe that the facial function outcomes of end-to-side HFA are like the outcomes of the classic type of HFA but with less lingual morbidity (5-11). 

The evaluation of the facial nerve function after surgery is usually performed using the House-Brackmann (HB) scoring system which categorizes the patients into grades I to VI based on the facial tone, symmetry, and movement (15). The current study evaluated the facial function and tongue morbidity after end-to-side HFA in patients with proximal facial nerve injury due to skull base surgery.

In an animal model study on the facial nerve regeneration on 12 rats, the subjects were divided into six groups that underwent facial nerve cutting and repairing by suturing (16). The rats were sacrificed and histologically studied at 1, 3, 5, 7, and 9 weeks after the surgery. Based on the results, 50% and 75% of functional recoveries were observed in animals within the 3^rd^ and 5^th^ weeks, as well as in the 9^th^ week, respectively. While the earliest factor that has been restored was the eye closure in this survey (and was more easily observable), the vibrissae movement restoration was the slowest item. Such observation is under that reported by other authors who emphasized that the partial improvement of eye closure is the earliest sign of neural recovery in rodents. 

## Materials and Methods

**Patients**

A total of 10 patients (4 males and 6 females) with proximal facial nerve injury during skull base surgery who underwent end-to-side HFA from April 2009 to March 2013 have been studied. As the proximal end of the facial nerve in the cerebello-pontine angle area had been loosed during these surgeries, the interposition nerve grafting have not been possible and they have undergone the nerve anastomosis. The patients’ age were within the range of 24-56 years old (average age: 44.5 years). All the subjects were reported with grade VI facial nerve paralysis following the excision of skull base pathologies. 

The etiology of the paralyzes was cholesteatoma (involving the internal auditory canal) in five patients, jugular paraganglioma in two patients, cerebellopontine angle tumor in two subjects, and squamous cell carcinoma of the temporal bone in one case (Table 1). In this study, six (60%) and four (40%) patients underwent anastomosis within the first year of paralysis (group A) and after one year (group B), respectively. In addition, six (60%) and four (40%) subjects suffered from the right-side and left-side paralysis, respectively.

**Table 1:** Descriptive information of patients and their final facial nerve functional status

No.	Gender	Age (year)	Duration of paralysis^a^	Final HB grade^b^	Paralysis site	Etiology
**1**	Male	24	A	3	Right	Cholesteatoma
**2**	Male	33	B	3	Right	Cholesteatoma
**3**	Male	46	A	3	Left	Vestibular schwannoma
**4**	Male	50	B	4	Left	Cholesteatoma
**5**	Female	56	B	4	Right	Jugular paraganglioma
**6**	Female	44	A	3	Right	Temporal tumor
**7**	Female	56	A	3	Right	Cholesteatoma
**8**	Female	40	A	4	Right	Cholesteatoma
**9**	Female	43	B	3	Left	Jugular paraganglioma
**10**	Female	53	A	3	Left	Vestibular schwannoma

a: A: anastomosis within 12 months of paralysis; B: anastomosis after 12 months of paralysis

b: House-Brackmann grading system

Surgical technique 

An approximately 8 cm incision was made which started at the lower third of the postauricular area (1 cm behind the sulcus and over the mastoid tip) and extended parallel to the sternocleidomastoid muscle at the anterior side. The anterior third of the attachment of the sternocleidomastoid muscle was cut to get exposure for the mastoid process and stylomastoid area. If the mastoid process has not been resected in the earlier temporal surgery for the elimination of disease, its partial resection for the evaluation of the facial nerve under the microscopic vision is mandatory. The mastoid part of the facial nerve (from the second genu to the stylomastoid foramen) has been skeletonized with the diamond burr and prepared for mobilizing from the bed. This part of the technique increases the length of the facial nerve for anastomosis. Chorda tympani was sectioned from the facial nerve, and the nerve was cut at the level of the second genu in a beveled cross-section. Then, the nerve could be mobilized up to pes anserinus and was ready. The preferred location of facial nerve cutting was around the first genu, and cable grafting was used if facial nerve cutting was mandatory in pes anserinus. By exposing the hypoglossal nerve behind the internal jugular vein at the level of the lateral process of the axis, it was proximally dissected as much as possible. An oblique incision was rostrally performed along the one half of the hypoglossal nerve diameter, just proximal to the branching of the descending hypoglossi.

The free end of the facial nerve approximated to the incised area of the hypoglossal nerve in a tension-free state. The epineurium of the facial nerve was sutured with 9-0 monofilament nylon in three or four points to the proximal surface of the incised hypoglossal nerve. Several approximations of the surrounding tissues were conducted by the sutures to decrease the tension of the anastomosis. After the hemostasis, the surgical incision was closed in two layers. 

All surgical anastomoses were performed by one surgeon. All the patients had follow-up visits for 18 months after the surgery. The data of the subjects were gathered from the hospital records, including pictures and movies. The assessment of the facial nerve function was based on the HB scoring system.

## Results

**Fig1:**
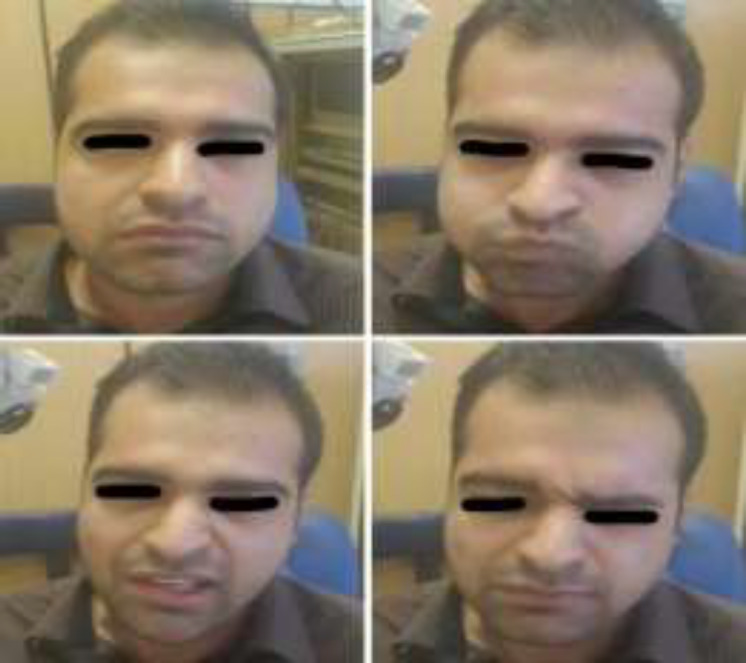
Facial nerve function in the end-to-side hypoglossal-facial anastomosis after 18 months

A total of 10 patients with iatrogenic proximal facial nerve paralysis due to skull base surgery who underwent end-to-side HFA entered into the study. All these patients had complete facial nerve palsy (grade VI of HB scoring confirmed by electromyographic studies). After 18 months of follow-up, seven (70%) and three (30%) patients were reported with grades III and IV based on HB scoring, respectively (Fig.1).

Among patients in group A (anastomosis within the first year of paralysis), one and five subjects were reported with grade III and IV, respectively. In contrast, in group B (anastomosis after the first year of paralysis), two patients had grade IV, and two subjects were reported with grade III. None of the cases showed tongue atrophy as a complication of the surgery. The end-to-side method of HFA can decrease the incidence of tongue atrophy in patients.

## Discussion

In order to recover the facial nerve function after paralysis due to iatrogenic proximal nerve injury, end-to-side HFA is an appropriate surgical approach. One of the advantages of this technique is the lower rate of surgical complications, such as tongue atrophy. When the surgeon is uncertain about the anatomical continuity of the facial nerve for spontaneous axonal regrowth, some authors prefer delaying surgery up to 2 years (17). However, when the nerve is anatomically disrupted, HFA should be performed for better functional results as soon as possible after injury (5,6,18). 

Another study has reported a direct relationship between the duration of paralysis and outcome in patients with longstanding facial paralysis (19). In a study by Slattery and the colleagues, they have studied 19 cases (18 patients with HB grade VI and 1 subject with HB grade V) and showed that after end-to-side HFA, 36.8%, 47.4%, and 15.8% of the cases were reported with HB grades III, IV, and V in a mean follow-up period of 4 years, respectively (10). All HB grade III patients had HFA within 6 months of injury. By the use of the same technique for 15 patients, another study reported 73%, 20%, and 7% of the subjects with grades III, IV, and V, with a median follow-up of 57.7 months, respectively. Less satisfactory results were observed when patients were managed in more than 2 years after nerve injury (9,11). In another series, 12 patients that five of them had HB grade V, six patients had HB grade VI, and the status of the last one was not scored, has been reported. After 12 months of follow-up following the use of the HFA technique, 70%, 20%, and 7% of the subjects were reported with grades III, IV, and V, respectively (20). 

In the largest series of 24 patients studied by Martins and the colleagues, 71% (n=17), 20% (n=6), and 4% (n=1) of the patients achieved grades III, IV, and V, respectively. He believed that the modified partial section of the hypoglossal nerve was as effective as classic hypoglossal-facial neurorrhaphy for facial reanimation^5^. In the present study, the patients who had anastomosis within the first year of paralysis showed better results (out of seven patients, five subjects with final grade III had the anastomosis within the first year of paralysis).

In a study by Samii et al., they followed the same procedure for 17 patients with HB grades V and VI. The mean time of anastomosis after the injury to the facial nerve was 18 months. In the aforementioned study, grades II, III, IV, and V were achieved in 5.8%, 76.5%, 11.7%, and 5.8% of the subjects, respectively (9). In the current study, after 18 months of follow-up, seven (70%) and three (30%) patients had HB grades III and IV, respectively, which is similar to the results of other studies (6,9,20). 

A retrospective study conducted by Mohamed and the colleagues on 22 patients with preoperative facial paralysis HB grade VI compared different techniques, namely facial nerve interposition graft, end-to-end HFA, jump graft, and end-to-side HFA for facial nerve reconstruction (21). The end-to-side HFA was performed on four patients (two primary and two secondary nerve anastomoses). With these four cases achieving HB grade III after the surgery, they concluded that direct end-to-side HFA was the best option in their series. However, Han et al. by the comparison of three surgical techniques (i.e., end-to-end, end-to-side, and split anastomoses) on 14 patients (seven, three, and four patients, respectively) concluded that split type HFA resulted in more favorable outcomes (14). 

The facial outcome was favorable (HB grades II and III) in 33.3% of end-to-side patients. In another study, the authors have concluded that no significant difference was observed between the two techniques, namely classic hypoglossal-facial neurorrhaphy and end-to-side HFA (6). In a comparative study carried out by Socolovsky and his colleagues on 77 patients with proximal facial nerve injury and preoperative HB grade VI, the best outcome (HB≤4) was observed in 92.2% of patients who had hemihypoglossal-facial direct neurorrhaphy that is side to end HFA (13). However, 54.5 % of the subjects who had hemihypoglossal-to-facial nerve transfer with an interposed sural nerve graft showed HB ≤ 4 improvement in contrast to 66.7 % of those undergoing the masseter-to-facial nerve transfer technique. 

In the above-mentioned study, it was concluded that the side to end procedure produces the most satisfactory facial reanimation results (13). Therefore, there is much debate about exact facial function findings after this procedure and even selection of facial reanimation surgical technique in proximal facial nerve injuries, especially after skull base procedures. According to the literature, there is satisfactory tongue function following HFA (5,7-11). In the current study, there was no evidence of tongue atrophy during the follow-up. After the HFA procedure, all the patients in the present study had HB grade III or IV and preservation of tongue movement.

## Conclusion

It seems that early end-to-side hypoglossal-facial anastomosis can be a favorable surgical option with good facial function results for reanimating the facial function in patients with the paralysis of the facial nerve after skull base surgery. Recommending to carry out further studies with larger sample size might be more informative. 
